# The impact of urine microbiota in patients with lower urinary tract symptoms

**DOI:** 10.1186/s12941-021-00428-9

**Published:** 2021-04-15

**Authors:** Hsiang-Ying Lee, Jiunn‐Wei Wang, Yung-Shun Juan, Ching-Chia Li, Chung-Jung Liu, Sung Yong Cho, Hsin-Chih Yeh, Kuang-Shun Chueh, Wen-Jeng Wu, Deng‐Chyang Wu

**Affiliations:** 1 Department of Urology, Kaohsiung Municipal Ta-Tung Hospital, Kaohsiung, Taiwan; 2 Graduate Institute of Clinical Medicine, College of Medicine, Kaohsiung Medical University, Kaohsiung, Taiwan; 3 Department of Urology, Kaohsiung Medical University Hospital, Kaohsiung, Taiwan; 4 Department of Urology, School of Medicine, College of Medicine, Kaohsiung Medical University, Kaohsiung, Taiwan; 5 Division of Gastroenterology, Department of Internal Medicine, Kaohsiung Medical University Hospital, Kaohsiung Medical University, Kaohsiung, Taiwan; 6 Regenetative Medicine and Cell Therapy Research Center, Kaohsiung Medical University, No.100, Tzyou 1st Rd., Sanmin Dist., Kaohsiung, 80756 Taiwan; 7 Department of Urology, Seoul National University Hospital, Seoul, Korea; 8 Department of Medicine, Faculty of Medicine, College of Medicine, Kaohsiung Medical University, Kaohsiung, Taiwan

**Keywords:** Urine microbiota, Benign prostate hyperplasia, Lower urinary tract symptoms

## Abstract

**Introduction:**

Inflammation and infection are causative factors of benign prostatic hyperplasia (BPH). Urine is not sterile, and urine microbiota identified by DNA sequencing can play an important role in the development of BPH and can influence the severity of lower urinary tract symptoms (LUTS).

**Materials and methods:**

We collected mid-stream voided urine samples from BPH patients and control participants and stored them in a freezer at − 80 °C. All enrolled participants were requested to provide information about their clinical characteristics and complete the International Prostate Symptom Score (IPSS) questionnaire. Each step of the procedure, including the extraction of the genomic DNA from the urine samples; the amplification by polymerase chain reaction (PCR); PCR product quantification, mixing, and purification; DNA library preparation; and sequencing was performed with quality control (QC) measures. Alpha diversity was indicative of the species complexity within individual urine samples, and beta diversity analysis was used to evaluate the differences among the samples in terms of species complexity. Pearson’s correlation analysis was performed to calculate the relationship between the clinical characteristics of the participants and the microbiota species in the urine samples.

**Results:**

We enrolled 77 BPH patients and 30 control participants who reported no recent antibiotic usage. Old age, high IPSS and poor quality of life were observed in the participants of the BPH group. No significant differences were observed in the alpha diversity of the samples. In the beta diversity analysis, there was a significant difference between the microbiota in the samples of the BPH and control groups according to ANOSIM statistical analysis. On comparing the groups, the ten bacterial genera present in the samples of the BPH group in descending order of abundance were: *Sphingomonas, Bacteroides, Lactobacillus, Streptococcus, Alcaligenes, Prevotella, Ruminococcaceae UCG-014, Escherichia_Shigella, Akkermansia,* and *Parabacteroides*. Spearman’s correlation analysis revealed that urine samples showing the presence of the bacterial genera *Haemophilus*, *Staphylococcus, Dolosigranulum, Listeria, Phascolarctobacterium, Enhydrobacter, Bacillus, [Ruminococcus]torques, Faecalibacterium, and Finegoldia* correlated with a high IPSS, and severe storage and voiding symptoms (*P* < 0.05).

**Conclusion:**

Our current study shows that dysbiosis of urine microbiota may be related to the development of BPH and the severity of LUTS. Further research targeting specific microbes to identify their role in the development of diseases is necessary and might provide novel diagnostic biomarkers and therapeutic options.

## Introduction

Benign prostatic hyperplasia (BPH) is one of the most common causes contributing to the lower urinary tract symptoms (LUTS) in elderly male patients, and its prevalence increases with age [1]. A previous meta-analysis revealed a prevalence of BPH in 26.2% of the participants tested [2]. LUTS can impact the quality of life and disturb routine activities. BPH is defined as the histological proliferation of connective tissue, smooth muscle, and epithelial cells, predominantly within the prostatic transition zone. Inflammation may increase the risk and severity of BPH and LUTS, although the underlying mechanism is unclear. Metabolic risk factors such as obesity induce inflammatory processes that are associated with the prevalence of BPH [3]. Systemic inflammation and oxidative stress induced by metabolic syndromes may lead to the BPH proliferative pathway [4]. Previous studies have discovered that the severity of the histological inflammatory conditions in the prostate specimen is associated with the extent of prostate enlargement [5]. Additionally, infection has been recognized as a factor related to the increased severity of BPH symptoms, while prior gonorrhea or prostatitis increases the possibility of occurrence of LUTS and the requirement of surgery for BPH [6, 7].

Human microbiota, which is the collective term for microorganisms in the human body comprising bacteria, viruses, parasites, and fungi, maintains symbiosis in the body, including the genitourinary tract. This influence of the human microbiota on human health and disease development is verified by emerging evidence. Dysbiosis, which is defined as an alteration in the normal composition of the microbiota, may be associated with diseases [8]. Previously, urine was considered sterile based on the results of the standard clinical culture tests. However, urine is no longer considered sterile, since the advancement of high-throughput DNA sequencing, by which bacteria can be detected in urine samples of even culture-negative healthy individuals [9–11]. Furthermore, alterations in the urine microbiota composition and in the environment were noted in various urologic diseases. By using 16S rRNA gene sequencing, previous studies have shown that the characteristics of urine microbiota are related to an increase in the episodes of urge urinary incontinence (UUI) in adult women [12–14]. The results obtained by 16S rRNA sequencing were also supported and complemented by enhanced urine culture methods although standard urine culture is negative [15–18]. Pearce et al. demonstrated that in nearly half of the sequence-positive samples in UUI patients, *Lactobacillus* and *Gardnerella* were the most common urotypes [14]. *Escherichia coli* and *Enterococcus* showed diversity in distribution between the samples of the patients with prostate cancer and BPH [19]. Therefore, ecological dysbiosis in urine may play an important role in the pathogenesis of prostate diseases [19]. In brief, urine microbiota may impact disease pathogenesis in both genders.

Standard culture-negative urine does not preclude the presence of urinary microbiota, which may still contribute to the development of BPH. To verify this possibility, we compared the composition of the urinary microbiota in BPH patients to that in non-BPH participants by 16S rDNA amplicon sequencing. According to recent Bajic et al. study, severity of LUTS is related to bladder microbiome [20]. In addition, we also investigated the relationship between the bacterial population in the urine samples and the clinical parameters in the participants, including the severity of LUTS, quantified using a questionnaire in the present study.

## Materials and methods

### Patient population and urine collection

All procedures involving human participants were reviewed and approved by the Kaohsiung Medical University Hospital Institutional Review Board (KMUHIRB-F(I)-20,190,013). Informed consent was obtained from all participants, and all experiments were performed in accordance with relevant guidelines and regulations. We recruited a cohort of patients with BPH, undergoing medical treatment for LUTS. Participants with other diseases that could interfere with voiding conditions, such as urinary tract infection, abnormal urinary tract anatomy, hereditary and congenital diseases, neurological diseases, malignancy, and history of spinal injury were excluded. Males without LUTS were enrolled as participants of the control group. Participants were also excluded if they had received an antibiotic treatment in the past two months, or a recent indwelling catheter. For each participant, mid-stream voided urine was collected with a sterile tube and stored in a freezer at − 80 °C. All participants were requested to provide basic information about their clinical characteristics, and complete the International Prostate Symptom Score (IPSS) questionnaire which includes a total of 8 questions: 7 symptom questions (score 0–5 points) and one quality of life due to urinary symptoms question (QOL, score 0–6 points) [20]. We also checked the laboratory data for all participants, including fasting blood glucose, hemoglobin A1c (HbA1c), cholesterol, triglyceride, low-density lipoproteins (LDL), high-density lipoproteins (HDL), glutamic oxaloacetic transaminase (GOT), glutamic pyruvic transaminase (GPT), blood urea nitrogen (BUN), creatinine, prostate specific antigen (PSA), free PSA, testosterone, and insulin. The size of the prostate of BPH patients was calculated using sonography. The clinical characteristics of the patients are shown in Table 1. For every participant, 10–20 ml urine sample were centrifuged at 14000 rpm for 15 min to collect the microbe. After centrifugation, supernatant discard and the pellet was resuspended with 500ul ST1 buffer (CatchGeneTM Stool DNA Kit content) for the cell lysis step according to the manuscript of CatchGene stool DNA extraction kit. Each step of the procedure, including the extraction of the genomic DNA; amplification by PCR; PCR product quantification, mixing, and purification; DNA library preparation; and sequencing, was performed with quality control (QC) measures. DNA was extracted from the precipitate using the CatchGeneTM Stool DNA Kit (CatchGene, Taiwan). For the negative control, double-distilled water (ddH_2_O) was used as the sample for the extraction process, and the eluate was further used as the negative control sample for the PCR amplification step. Specific primers (319 F: 5′-CCTACGGGNGGCWGCAG-3′, 806 R: 5′-GACTACHVGGGTATCTAATCC-3′) were used to perform the PCR amplification of 16S rDNA over the V3-V4 regions according to the 16S Metagenomic Sequencing Library Preparation procedure (Illumina). The indexed PCR product quality was assessed using the Qubit 4.0 fluorometer (Thermo Scientific) and Qsep100™ system. An equal amount of the indexed PCR product was mixed to generate the sequencing library. Finally, the library was sequenced on the MiSeq platform (Illumina), and paired-end 300-bp reads were generated. The raw data were merged and filtered to obtain clean data. Operational taxonomic unit (OTU) clusters were obtained from the data.

**Table 1:** Comparisons of demographic, clinical characteristics and bacteria alpha diversity between BPH group and normal control group

	BPH (n = 77)	Control (n = 30)	*P* value
Demographic			
Age	69.44 ± 8.23	61.97 ± 8.32	< 0.001^a^
Body mass index	24.17 ± 3.33	24.55 ± 2.87	0.616
Clinical Characteristics			
Fasting blood glucose	110.89 ± 17.05	114.48 ± 26.77	0.436
Prostate specific antigen (PSA)	3.02 ± 3.21	2.37 ± 3.94	0.406
Free PSA	0.60 ± 0.11	0.69 ± 1.25	0.613
Hemoglobin A1c (%)	5.81 ± 0.67	5.88 ± 0.53	0.660
Creatinine	0.98 ± 0.22	0.93 ± 0.18	0.393
BUN	15.44 ± 4.94	13.22 ± 2.93	0.036
HDL	53.93 ± 24.43	47.91 ± 11.76	0.239
Cholesterol	181.51 ± 42.61	182.28 ± 28.15	0.933
LDL	110.61 ± 33.82	116.75 ± 26.46	0.409
Testosterone	551.68 ± 246.50	540.75 ± 158.96	0.836
GOT	25.96 ± 7.63	26.50 ± 7.79	0.804
GPT	26.133 ± 12.86	30.56 ± 20.82	0.210
TG	108.68 ± 66.63	102.52 ± 44.11	0.667
IPSS	6.25 ± 4.19	2.16 ± 1.37	< 0.001^a^
Quality of life scores	2.09 ± 0.68	1.2 ± 0.41	< 0.001^a^
Parameter of Bacterial Alpha Diversity			
Observed species	256.17 ± 65.29	254.70 ± 51.58	0.912
Chao1	284.40 ± 65.63	288.21 ± 63.05	0.786
ACE index	277.65 ± 64.78	280.21 ± 57.85	0.850
Shannon index	5.81 ± 1.04	5.59 ± 0.90	0.314
Simpson index	0.93 ± 0.08	0.92 ± 0.06	0.697

^a^IPSS: International Prostate Symptom *Score*

### Statistical analysis

We used Student’s *t*-test for continuous variables to evaluate the differences in the clinical characteristics between the participants of the BPH and control groups. For urine microbiota analysis, we tested the significance of community composition and structural differences between the groups using ANOSIM. Welch’s *t*-test was used to identify the significant differences in species between the groups (*P* < 0.05) at various taxon levels, including the phylum, class, order, family, genus, and species. Pearson’s correlation analysis was performed to calculate the relationship between the clinical characteristics of the participants and the microbiota species in the urine samples. SPSS (Statistical Product and Service Solutions, version 22) software was used to analyze the data, and *P* values < 0.05 were considered statistically significant.

### Bioinformatic analysis

By using the FLASH (Fast Length Adjustment of Short Reads, v.1.2.11) software, we assembled the 300 bp paired-end raw reads derived from 16S rDNA amplicon sequencing [21]. For each representative sequence, the RDP (Ribosomal Database Project, v.2.2) classifier algorithm was employed to annotate taxonomy classification based on the information retrieved from the Silva Database v.132 [22]. Subsequent analysis of alpha and beta diversities was performed using the normalized data. Alpha diversity was indicative of the species complexity within individual urine samples based on six different criteria output from the QIIME (Quantitative Insights Into Microbial Ecology) pipeline, including observed OTUs, and Chao-1, Shannon, Simpson, ACE (Abundance-based Coverage Estimator), and Good’s coverage indices [23]. Beta diversity analysis was used to evaluate the differences among the samples in terms of species complexity. Two beta diversity parameters, the weighted and unweighted UniFrac [24], were calculated using the QIIME pipeline. Principal coordinate analysis (PCoA) was performed to acquire principal coordinates for the visualization of sophisticated and multidimensional data. PCoA analysis was conducted using the WGCNA (Weighted Correlation Network Analysis), stat, and ggplot2 packages in R software (v.2.15.3). Non-metric multidimensional scaling (NMDS) analysis was performed to fit the nonlinear model in ecological datasets by using the vegan package in R software (v.2.15.3) [25, 26]. The significance of all species among groups at various taxonomic levels was detected using differential abundance analysis with a zero-inflated Gaussian (ZIG) log-normal model as implemented in the “fitFeatureModel” function of the metagenomeSeq package of the Bioconductor software [27]. Statistically significant biomarkers were identified using LEfSe (linear discriminant analysis effect size) method [28]. Taxa with an LDA (linear discriminant analysis) score (log 10) > 4 were considered significant [27].

## Results

### Demographic and clinical characteristics of the participants

**Fig. 1:**
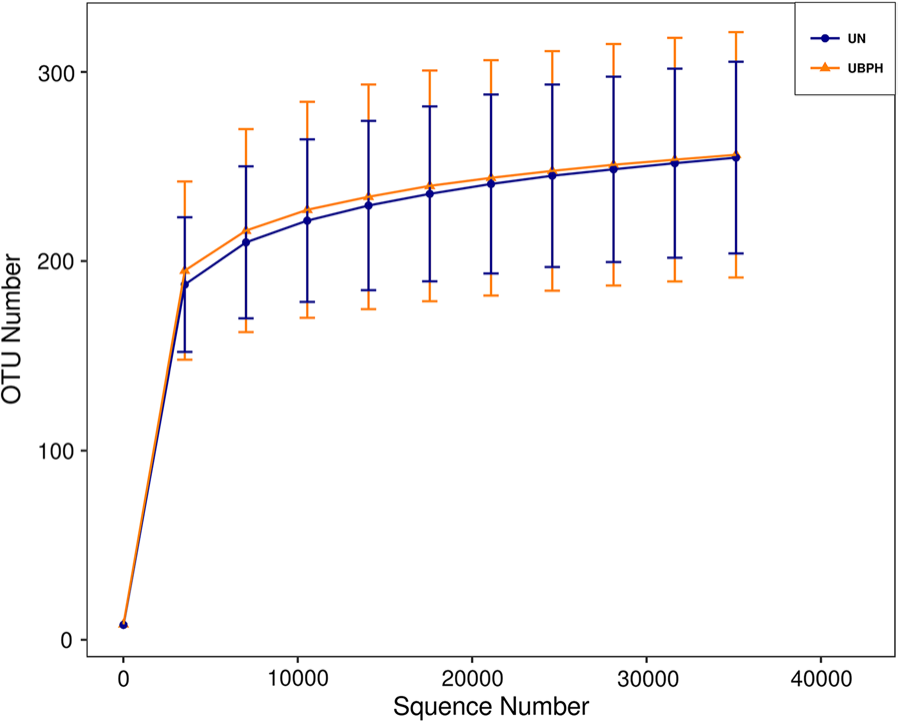
Rarefaction curve: The curve becomes flatter which means almost samples have been taken

We enrolled 77 BPH patients and 30 control participants into two groups in our microbiota analysis. All urine samples were collected with sterile tubes and provided adequate sequencing reads, named as UBPH and UN. This can be verified from the Rarefaction curve, which became a smooth reflection of reasonable species richness in Fig. 1. Detailed comparisons between the BPH and control groups are shown in Table 1. IPSS, QOL and age were significantly different between the participants of the two groups. More severity of LUTS, poor quality of life, and older age were seen in the patients of the BPH group than in the participants of the control group (IPSS for BPH group: 6.25 ± 4.19, IPSS for control group: 2.16 ± 1.37; QOL score for BPH group: 2.09 ± 0.68, QOL score for control group: 1.2 ± 0.41; age for BPH group: 69.44 ± 8.23, age for control group: 61.97 ± 8.32). No significant differences were observed in other clinical characteristics of the participants of both groups.

### Bioinformatic analysis

**Fig. 2:**
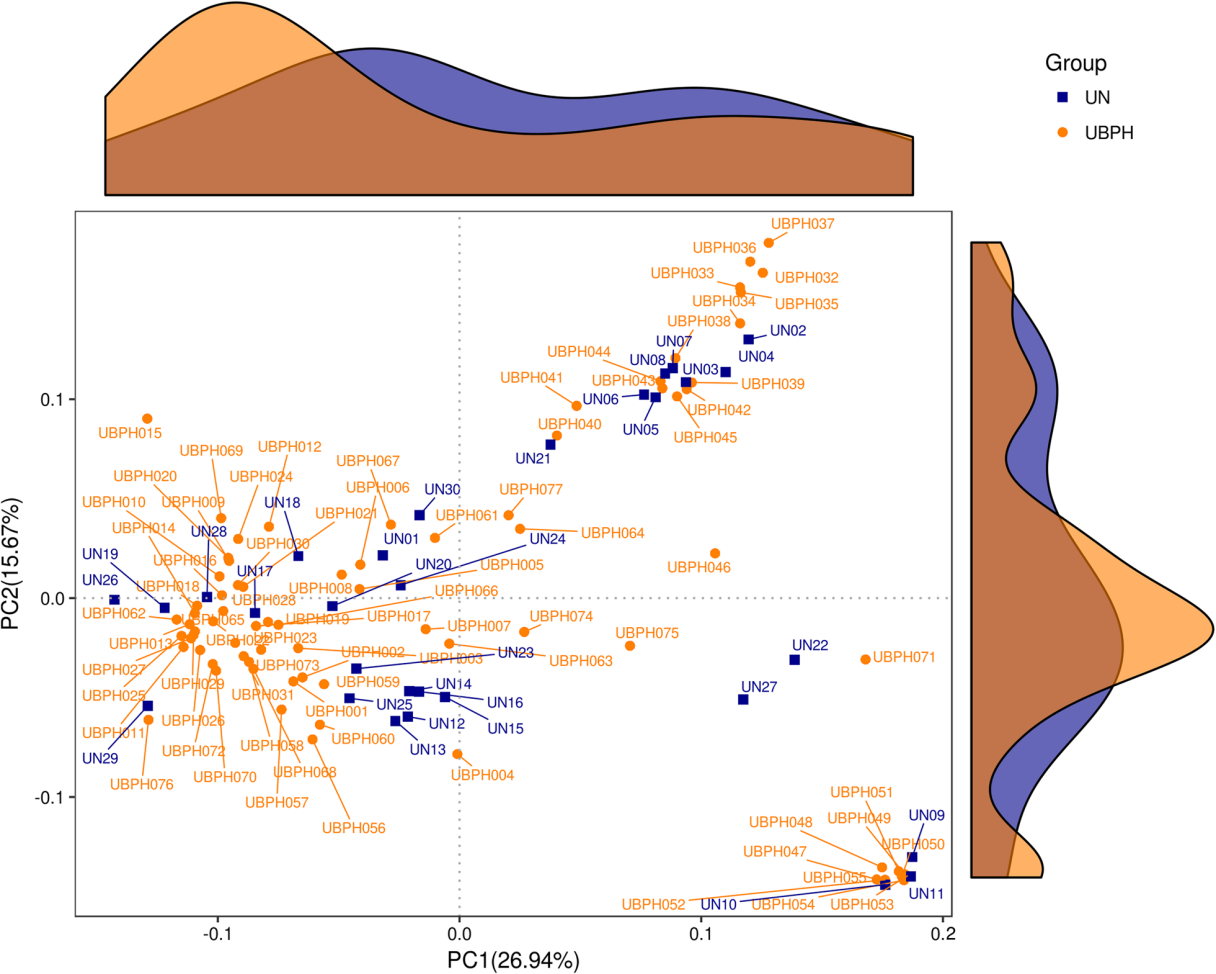
Principal Co-ordinates Analysis (PCoA): urine microbiota in both group clustered separately in PCoA plots

**Fig. 3:**
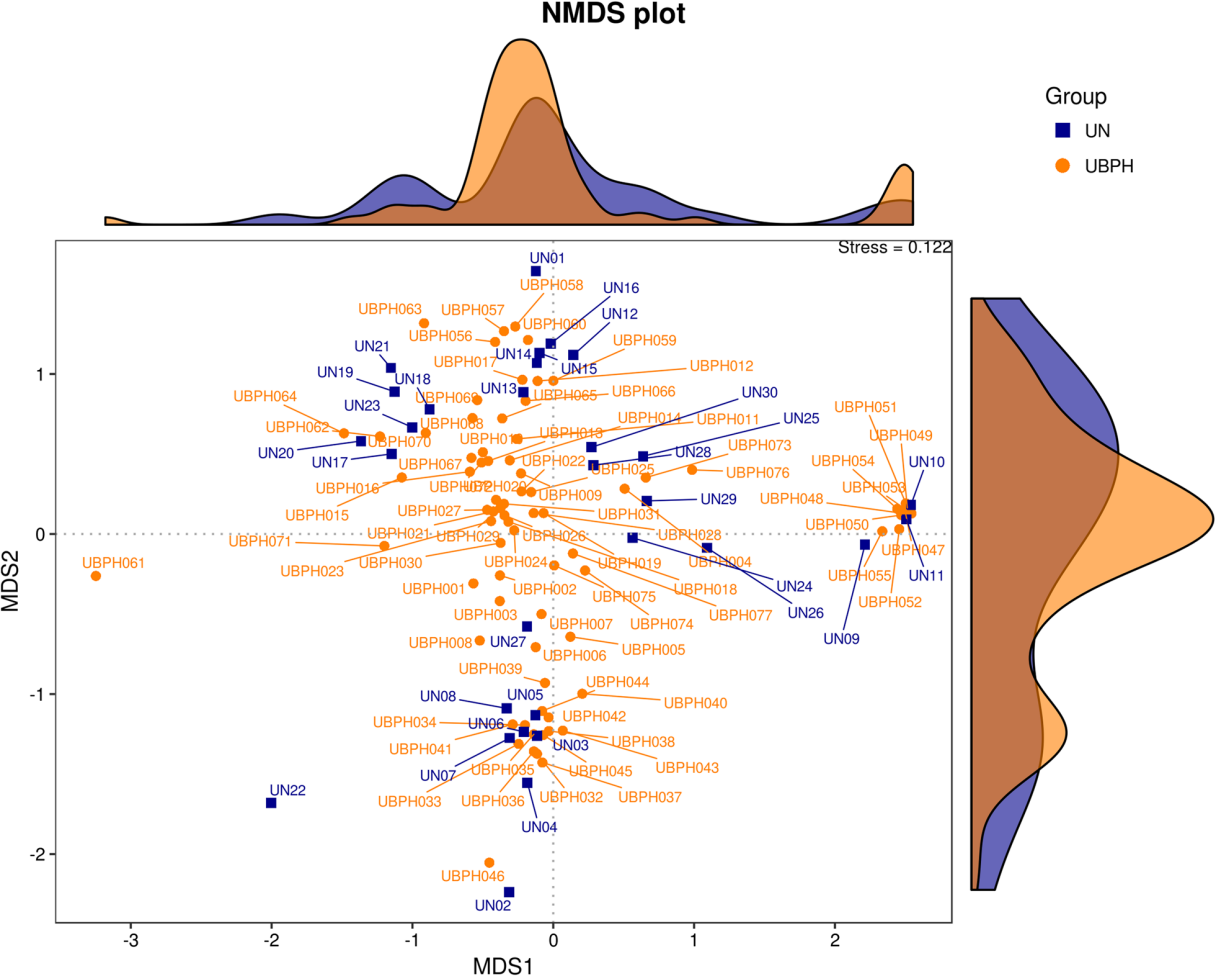
Non-metric Multidimensional Scaling (NMDS): non-linear model showed separate distribution between both groups

**Fig. 4:**
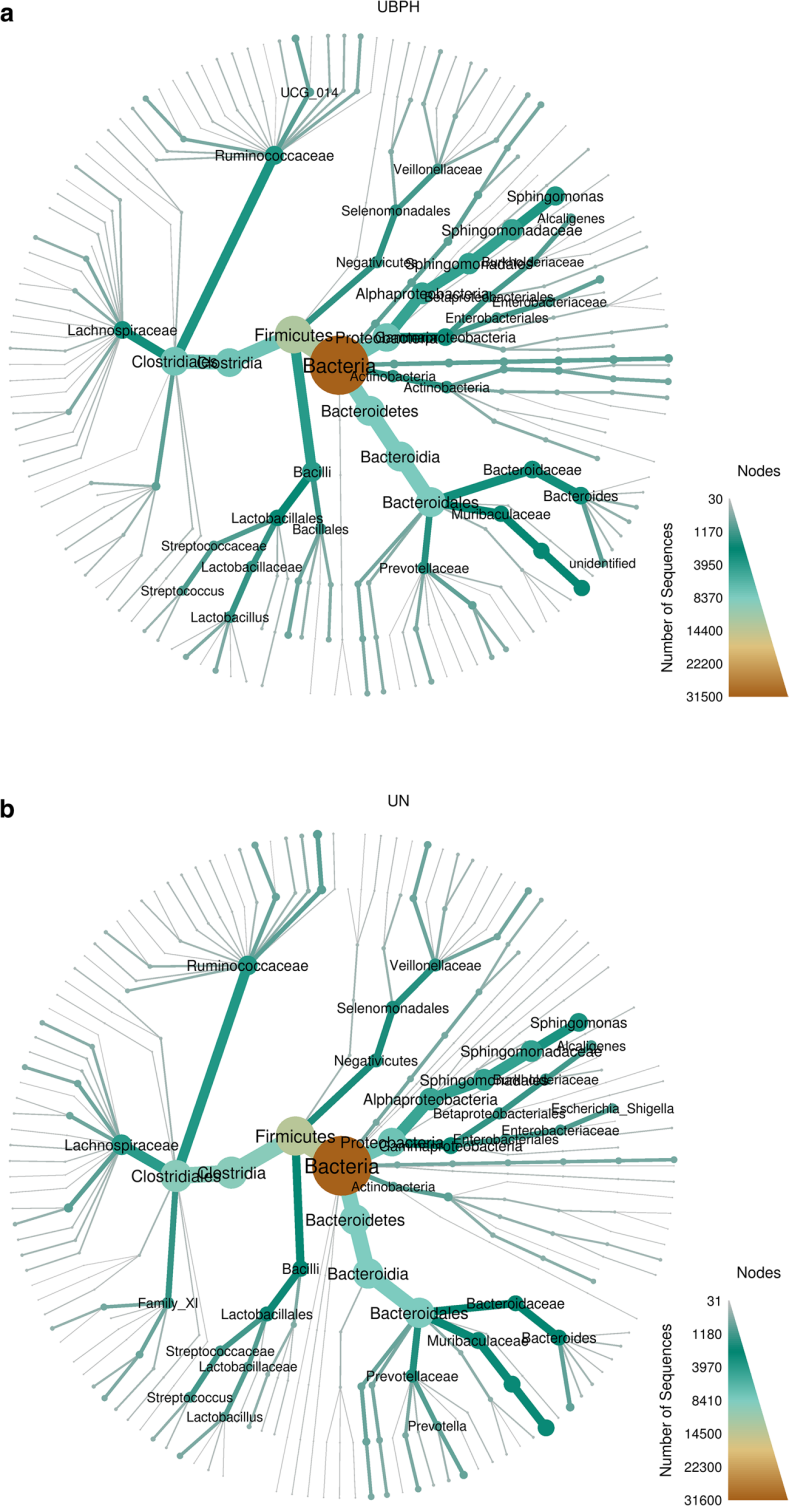
Heat tree: it presents richness of different taxonomy level by node size, edge thickness and color in (**a**): BPH group and (**b**): normal control group

From the Venn diagram, 2617 OTUs were identified in the samples of the BPH group and 1820 OTUs were identified in the samples of the control group. The groups had 1381 OTUs in common. Comparisons of alpha diversity between the samples of the BPH and control groups are also seen in Table 1. There were no significant differences between the BPH and control groups in terms of observed species (256.17 ± 65.29 vs. 254.70 ± 51.58), Chao-1 index (284.40 ± 65.63 vs. 288.21 ± 63.05), ACE index (277.65 ± 64.78 vs. 280.21 ± 57.85), Shannon index (5.81 ± 1.04 vs. 5.59 ± 0.90) and Simpson index (0.93 ± 0.08 vs. 0.92 ± 0.06). As shown in Fig. 2 through PCoA, we found urinary microbiota in BPH patients was distinct from that of control group. Statistically differences were observed after t-test analysis distance metrics (*P* < 0.001, *P* < 0.001 for weighted UniFrac, unweighted UniFrac). A similar distribution can also be found in the non-metric multidimensional scaling (NMDS) shown in Fig. 3. We compared the differences between the samples in the form of a heat tree. The circular heat tree represents the richness of different taxonomy levels by node size, edge thickness, and color (Fig. 4a, b).

**Fig. 5:**
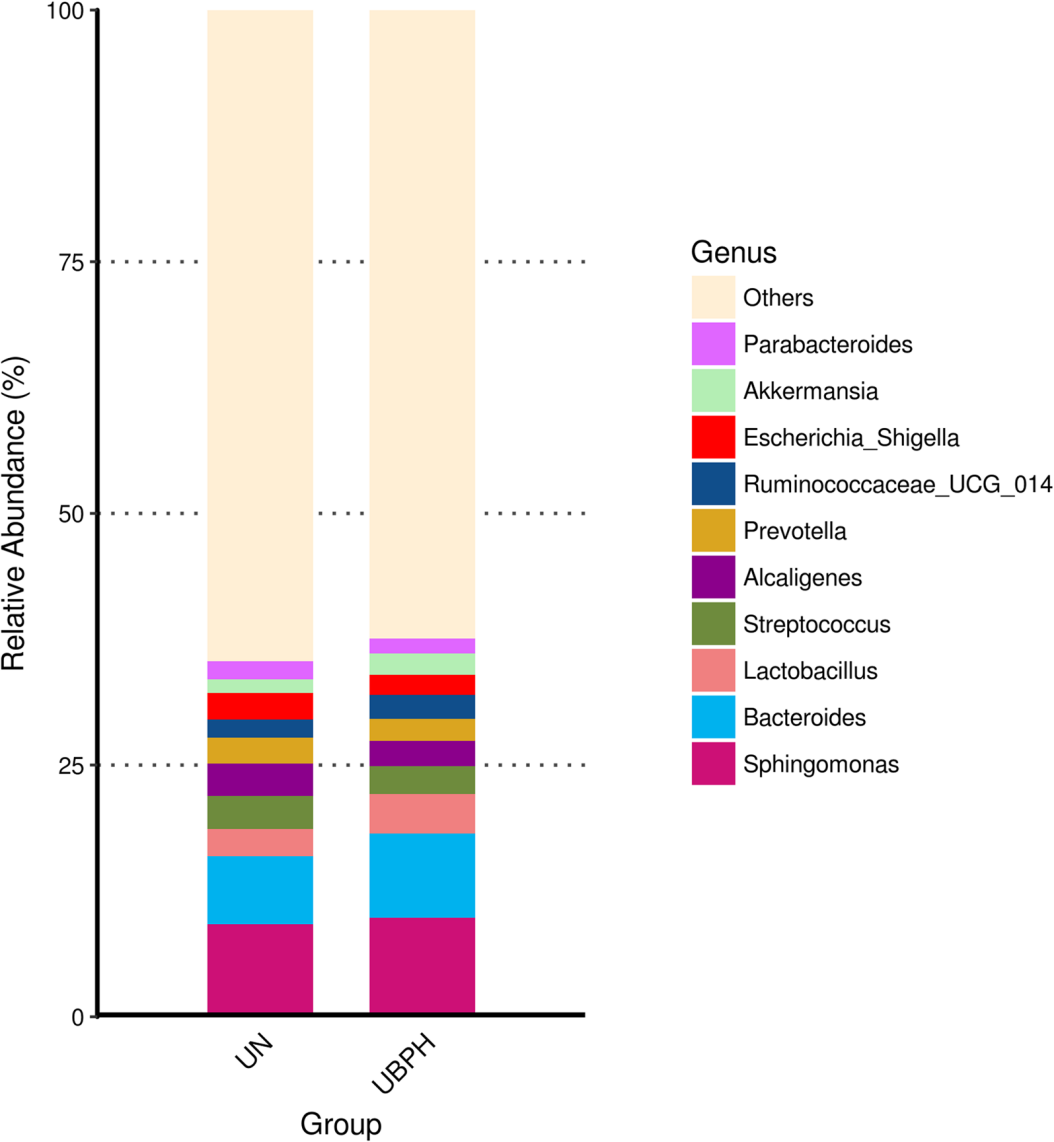
Top 10 abundant species comparing BPH and control group at the genus level

**Fig. 6:**
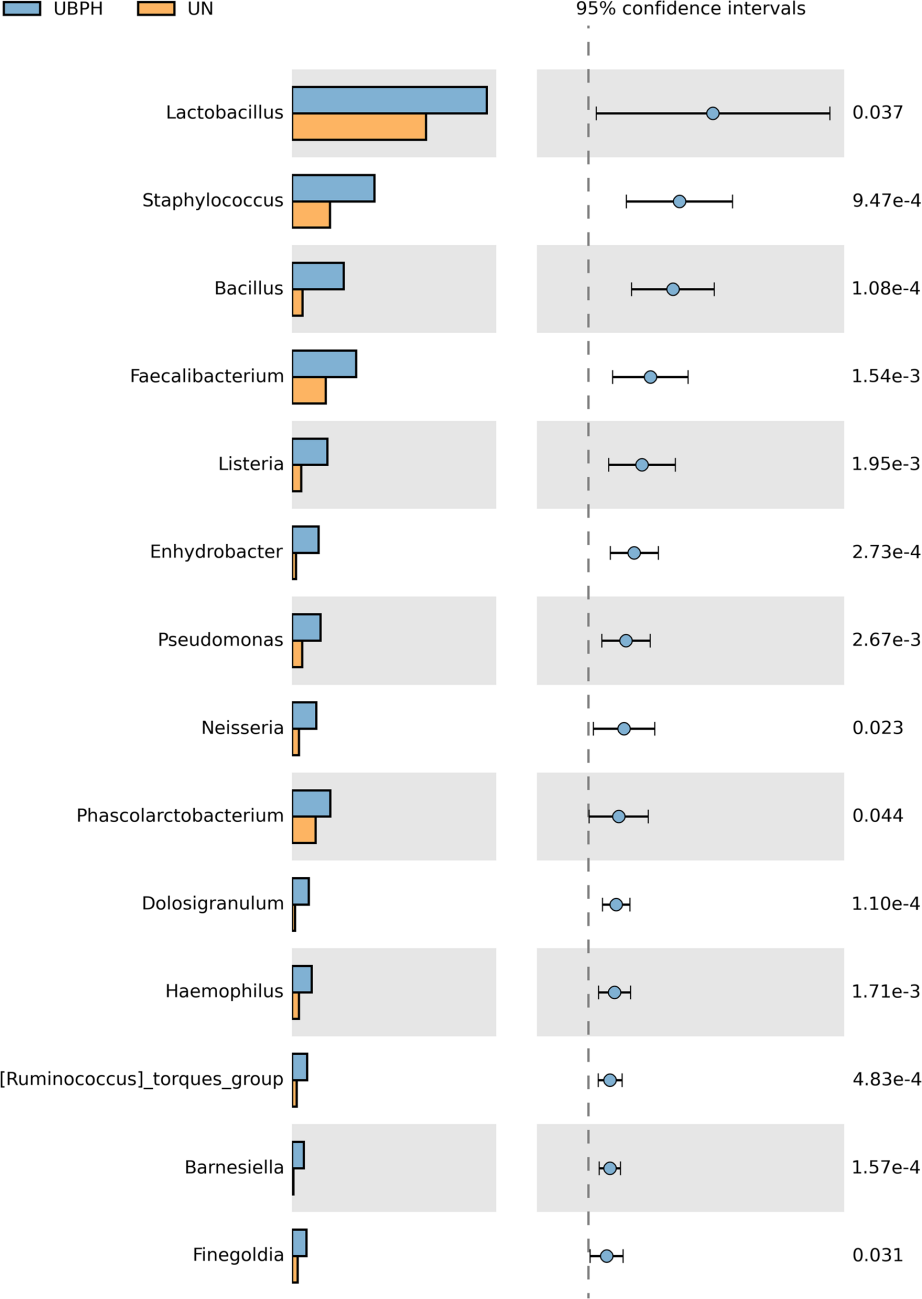
The bar graph revealed most top 15 significant expression in the BPH group at the genus level using statistical analysis of metagenomic profiles (STAMP) to perform Welch's t-test

Based on the results of species annotation, we identified the 10 most abundant species at genus level to form the distribution histogram of relative abundance in Fig. 5. The top ten bacterial genera in descending order of abundance in the urine samples were *Sphingomonas, Bacteroides, Lactobacillus, Streptococcus, Alcaligenes, Prevotella, Ruminococcaceae UCG-014, Escherichia_Shigella, Akkermansia,* and *Parabacteroides*. From the results of ANOSIM statistical analysis in Table 2, a significant difference was observed between the BPH and control groups (*P* = 0.01). STAMP (Statistical analysis of metagenomic profiles) software was used to perform Welch’s *t*-test and discover the significant difference between the groups at the genus level, as shown in Fig. 6. The bar graph reveals that the 15 genera which were significantly expressed in the samples of the BPH group, are *Lactobacillus, Staphylococcus, Bacillus, Faecalibacterium, Listeria, Enhydrobacter, Pseudomonas, Neisseria, Phascolarctobacterium, Dolosigranulum, Haemophilus, [Ruminococcus]torques, Bamesiella, Finegoldia,* and *Prevotellaceae NK3B31* group.

**Table 2:** Anosim statistic analysis: comparing the difference of the composition of microbiota between BPH group and normal control group

Group	R-value	P-value
UN-UBPH	0.115	0.01

### Correlation analysis

**Fig. 7:**
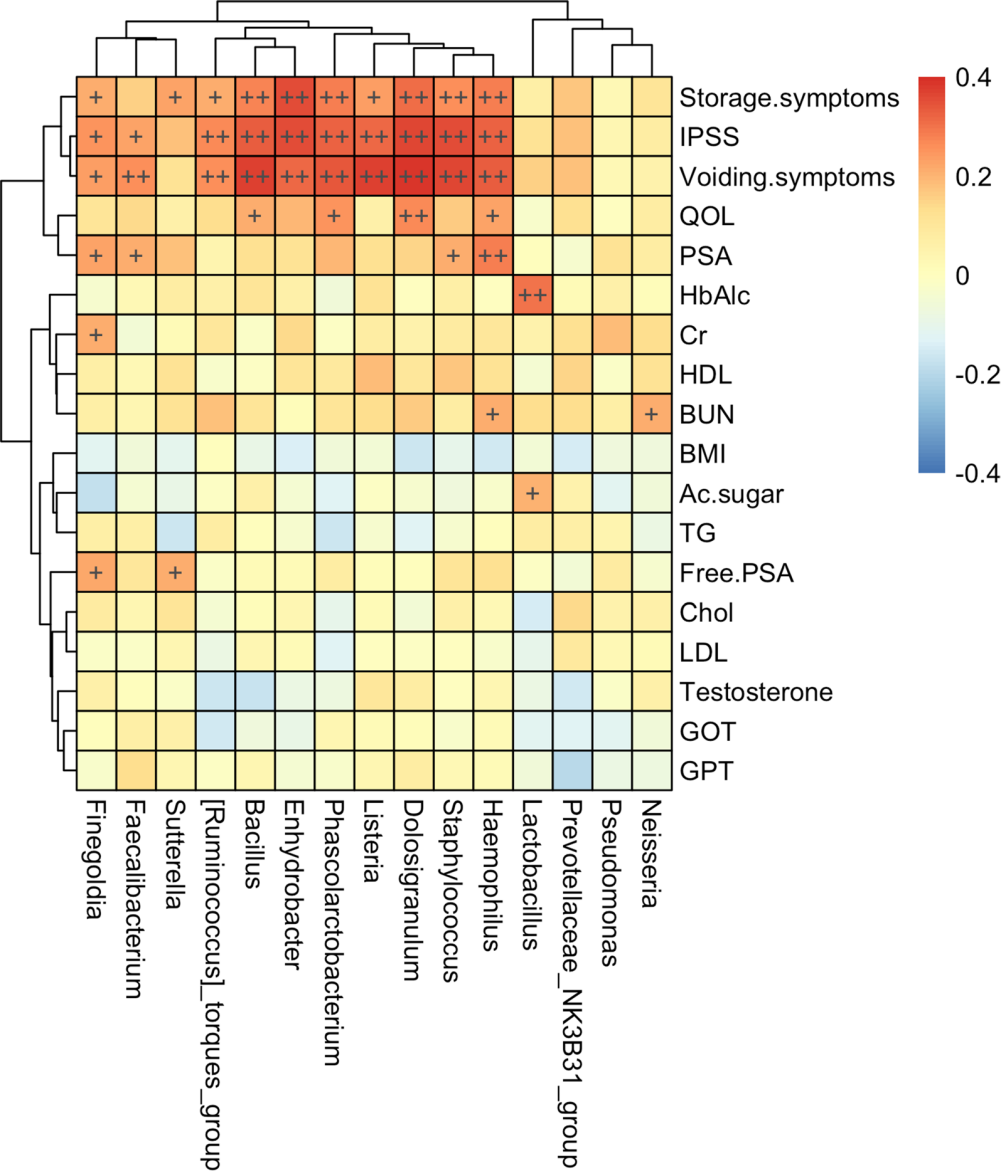
Spearman correlation analysis: identify significant correlation between specific microbes and clinical characteristics

In Fig. 7, Spearman’s correlation analysis revealed that urine samples with *Haemophilus*, *Staphylococcus, Dolosigranulum, Listeria, Phascolarctobacterium, Enhydrobacter, Bacillus, [Ruminococcus]torques, Faecalibacterium, and Finegoldia* presented high IPSS, and severe storage and voiding symptoms (*P* < 0.05). The urine samples with a higher abundance of *Haemophilus* showed higher PSA levels while the presence of *Lactobacillus* in urine samples (*P* < 0.05) was positively associated with high HbA1c and glucose levels.

## Discussion

Since the importance of microbiota was established, numerous studies have addressed the impact of microbiota and the balance between host and resident microbiota on the human body [11, 29]. In addition to gut microbiota, urine microbiota also impacts urinary diseases, but is poorly understood. In this study, we describe the urinary microbiota in males with and without BPH, and establish the association between abundance of species and clinical characteristics of participants, using 16S rDNA amplicon sequencing. To our knowledge, few studies have discussed the detailed impact of urinary microbiota in BPH patients. It was incorrectly considered that urine is sterile, primarily on the basis of culture-dependent methods. However, microbes exist in urine of adult men and women even without clinical infection [10, 16].

In a study by Lewis et al. [9], 16S rRNA sequencing on mid-stream voided urine samples revealed that the number and diversity of microbiota change with age in healthy, asymptomatic individuals of both genders. It is assumed that these changes may be associated with the occurrence of BPH with aging, in men [30]. Although both BPH and LUTS are highly prevalent in men and increase in prevalence with age, only 25–50% of patients with BPH suffer from significant LUTS, while only half to two-thirds of patients with LUTS showed bladder outlet obstruction (BOO) on urodynamic testing [31]. In addition, the difference in individual response suggests that the pathophysiology of BPH is heterogeneous and poorly understood. The diversity within BPH participants also can be demonstrated in our results. The causes of BPH/LUTS are multifactorial, and inflammation is one of the important factors that was proposed on observation of chronic inflammation coexisting with histologic changes in resected prostate specimen in BPH [32]. Prostate inflammation may be triggered by bacterial infection, followed by the secretion of cytokines, chemokines, and growth factors including CD4, CD8, CD45, CD68, C-reactive protein, tumor necrosis factor, interleukin-6, and others [33–35]. Due to the above hypothesis, a link between BPH and microbiota was considered and a target for its diagnosis and treatment was set.

LUTS is composed of voiding and storage symptoms, which were mainly evaluated by IPSS. Clinically, we can stratify mild to severe symptoms with a symptom score. Bajic et al. [20] observed a distinct presence of microbiota in severe symptomatic patients who needed surgical therapy, and in minimally symptomatic controls. Compared to the study by Bajic et al., our results detected statistically significant differences in the relative abundance of specific microbes in the urine samples of BPH patients compared to the samples of the control group. Further evaluation of the urine microbiota elucidates association of some microbes with degrees of LUTS as well as with the voiding and storage symptoms. Significant differences in microbes were also present in this subset of symptoms. Nevertheless, it is hypothesized that dysbiosis of the urine microbiota may play an important role in the progression of BPH and LUTS. Within pathogens that over-represented in urine samples of BPH patients, *Haemophilus*, *Staphylococcus, Dolosigranulum, Listeria, Phascolarctobacterium, Enhydrobacter, Bacillus, [Ruminococcus]torques, Faecalibacterium,* and *Finegoldia* are associated with more severe LUTS.

*Haemophilus* spp. is reported as a rare urinary tract pathogen but needs to be investigated in patients with urinary tract abnormalities such as urolithiasis [36]. A previous study demonstrated that *Faecalibacterium* was more abundant in patients with diabetes mellitus (DM) and hyperlipidemia, compared to patients with only DM, as seen in the study by Fengping Liu et al. They also demonstrated that *Faecalibacterium* is associated with incidence of UUI. In addition, *Bacillus* is also common in patients with DM, hypertension and hyperlipidemia [37]. After culture-independent 16S rDNA gene sequencing, *Finegoldia* was identified in urine samples from patients with severe preoperative urinary symptoms, undergoing surgery for pelvic organ prolapse and stress incontinence [38]. The presence of *Lactobacillus* was positively associated with HbA1c and glucose levels in our study as well as in the study by Jiawei Chen et al. Their results suggested that changes in the microbial community, specifically in *Lactobacillus*, may be an impact factor in LUTS [39]. According to previous studies, microbes can be identified in culture-negative urine.

In order to decrease the possibility of urine contamination, we collected mid-stream voided urine samples with sterile containers. Furthermore, voided urine is less easily contaminated with microbes in males than in females. Although collecting urine by sterile catheterization was an alternative method for our study, it was considered invasive, uncomfortable and not clinically feasible. Different urine collection methods show different urine microbiota. This may be due to differences between the microbiome of the urethra and the bladder [40]. According to this hypothesis, results of the urine microbiota in our study may represent a mixture of bacteria from the urethra and the bladder. Because antibiotic treatment can impact the composition of urine microbiota and the influence may persist for at least two months [41], we excluded participants who had taken antibiotics within the last two months.

Our study has some limitations. First, it is a cross-sectional study in which the cause-effect relationship is difficult to define between clinical characteristics and bioinformatics indicators. Therefore, animal experimentation is needed to clarify the role of specific microbes in the development of BPH and LUTS. Second, we only analyzed urine microbiota from voided urine; further work would include catheterized urine collection to identify the different microbes residing in the bladder and urethra. Furthermore, we did not analyze the relationship between the medications for BPH and urine microbiota. Thus, prospective studies need to clarify if different strategies will disturb the environment of the microbiota. Finally, another weakness is the lack of replicates, other cohort to verify the composition of urinary microbiota in BPH patients is justified.

## Conclusion

We have outlined the bacterial microbiota in the urine of BPH patients is different from that seen in control participants but some may be indistinguishable from control group. We also discovered that urine microbiota is significantly associated with the severity of LUTS, even in a subset of symptoms, and confirmed what Bajic et al. have demonstrated. The results suggest that some specific microbes may have important roles in the development and progression of BPH. Further research is needed to target these microbes for analyzing the pathobiology of BPH and improving LUTS.

## Data Availability

Not applicable.
